# Ovarian Blood Sampling Identifies Junction Plakoglobin as a Novel Biomarker of Early Ovarian Cancer

**DOI:** 10.3389/fonc.2020.01767

**Published:** 2020-09-25

**Authors:** Florian Weiland, Noor A. Lokman, Manuela Klingler-Hoffmann, Thomas Jobling, Andrew N. Stephens, Karin Sundfeldt, Peter Hoffmann, Martin K. Oehler

**Affiliations:** ^1^Adelaide Proteomics Centre, The University of Adelaide, Adelaide, SA, Australia; ^2^Institute for Photonics and Advanced Sensing, The University of Adelaide, Adelaide, SA, Australia; ^3^Department of Microbial and Molecular Systems (M^2^S), Laboratory of Enzyme, Fermentation and Brewing Technology (EFBT), KU Leuven, Leuven, Belgium; ^4^Discipline of Obstetrics and Gynecology, Adelaide Medical School, Robinson Research Institute, The University of Adelaide, Adelaide, SA, Australia; ^5^Future Industries Institute, University of South Australia, Adelaide, SA, Australia; ^6^Department of Gynecological Oncology, Monash Medical Centre, Clayton, VIC, Australia; ^7^Centre for Cancer Research, Hudson Institute of Medical Research, Clayton, VIC, Australia; ^8^Department of Molecular and Translational Sciences, Monash University, Clayton, VIC, Australia; ^9^Department of Obstetrics and Gynecology, Sahlgrenska Cancer Center, Institute of Clinical Science, Sahlgrenska Academy, University of Gothenburg, Gothenburg, Sweden; ^10^Department of Gynecological Oncology, Royal Adelaide Hospital, Adelaide, SA, Australia

**Keywords:** junction plakoglobin, ovarian cancer, early diagnosis, biomarker, proteomics, abundance protein depletion, saturation labeling 2D-DIGE, mass spectrometry

## Abstract

Ovarian cancer is the most lethal gynecologic malignancy. Early detection would improve survival, but an effective diagnostic test does not exist. Novel biomarkers for early ovarian cancer diagnosis are therefore warranted. We performed intraoperative blood sampling from ovarian veins of stage I epithelial ovarian carcinomas and analyzed the serum proteome. Junction plakoglobin (JUP) was found to be elevated in venous blood from ovaries with malignancies when compared to those with benign disease. Peripheral plasma JUP levels were validated by ELISA in a multicenter international patient cohort. JUP was significantly increased in FIGO serous stage IA+B (1.97-fold increase; *p* < 0.001; *n* = 20), serous stage I (2.09-fold increase; *p* < 0.0001; *n* = 40), serous stage II (1.81-fold increase, *p* < 0.001, *n* = 23) and serous stage III ovarian carcinomas (1.98-fold increase; *p* < 0.0001; *n* = 34) vs. normal controls (*n* = 109). JUP plasma levels were not increased in early stage breast cancer (*p* = 0.122; *n* = 12). In serous ovarian cancer patients, JUP had a sensitivity of 85% in stage IA+B and 60% in stage IA-C, with specificities of 76 and 94%, respectively. A logistic regression model of JUP and Cancer Antigen 125 (CA125) revealed a sensitivity of 70% for stage IA+B and 75% for stage IA-C serous carcinomas at 100% specificity. Our novel ovarian blood sampling – proteomics approach identified JUP as a promising new biomarker for epithelial ovarian cancer, which in combination with CA125 might fulfill the test criteria for ovarian cancer screening.

## Introduction

Ovarian cancer (OC) accounts for an estimated 239,000 new cases and 152,000 deaths worldwide each year (1). The high mortality rate of ovarian cancer arises due to the asymptomatic progression of the disease, resulting in over 70% of cases being diagnosed at advanced stage [International Federation of Gynecology and Obstetrics (FIGO) stage III and IV] when the cancer has spread to the abdominal cavity or to other organs. Detection of OC at an early stage, i.e., when it is still confined to the ovary (FIGO stage I), is associated with a 5-year survival rate of over 90% compared to <30% for women presenting with advanced disease (2, 3). Therefore, the detection of OC at an early stage is the most effective way to improve survival. However, at present no clinically applicable early detection test is available, and population screening is therefore not possible.

CA125, the current gold standard protein biomarker in OC, is only clinically approved to distinguish benign from malignant ovarian lesions and to assess tumor burden. Elevated levels of CA125, however, are only observed in <50% of early stage patients and CA125 is therefore not a useful tumor marker for early ovarian cancer detection (4).

Significant efforts have been undertaken to develop a biomarker-based early detection test for many years. However, a major “quantitative” challenge of OC biomarker research is to identify the cancer at an early stage when it is small and only secretes a very small amount of cancer-specific biomarker into the blood stream. Another significant “qualitative” problem of biomarker detection is that highly abundant proteins tend to obscure the detection of potential biomarkers that are usually in lower concentrations in biofluids such as serum or plasma (5).

To overcome the challenges of OC biomarker detection, we developed a novel approach of combining: (a) Ovarian blood sampling, to obtain blood with higher biomarker concentration downstream of the cancer, with (b) Abundance protein depletion and saturation labeling 2D-DIGE, to identify less abundant proteins in plasma and serum. Our strategy resulted in the identification of a promising new marker for epithelial ovarian cancer, junction plakoglobin (JUP, plakoglobin, γ-catenin).

JUP is a member of the Armadillo family of proteins and responsible for the linking of classical cadherins to α-catenin (6–9). It interacts with the desmosomal cadherins desmoglein-1 and desmocollin (10), anchoring cell-cell adhesion receptors into the cytoskeleton. JUP has never been described as diagnostic biomarker for OC or any other malignancy.

## Materials and Methods

### Patient Samples

**Figure 1:**
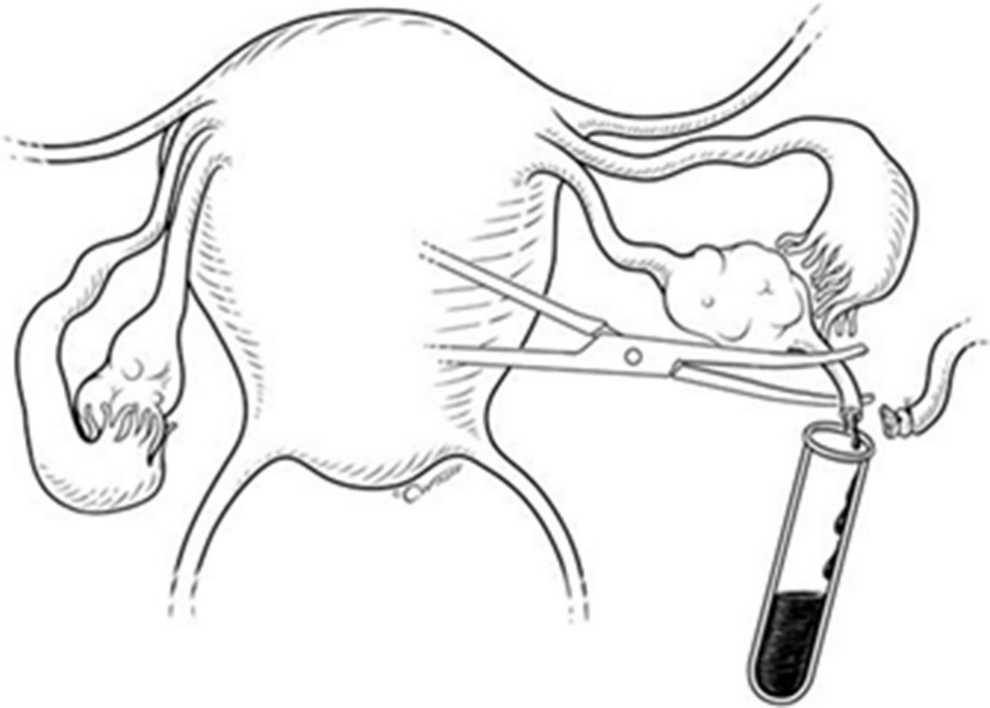
Ovarian blood sampling technique during total abdominal hysterectomy and bilateral salpingo-oophorectomy. Reproduced from Fogle RH, Stanczyk FZ, Zhang X, Paulson RJ. Ovarian androgen production in postmenopausal women. J Clin Endocrinol Metab. 2007; 92:3040–3, with permission of the Endocrine Society.

Blood from ovarian and peripheral (cubital) veins was collected intraoperatively with patient consent and approval by the Research Ethics Committee at the Royal Adelaide Hospital, Adelaide, South Australia (Protocol #080304) (Figure 1). Ovarian serum samples from 6 patients with stage IA high grade ovarian cancer (2 serous papillary and 4 endometrioid adenocarcinomas), 2 patients with functional ovarian cysts and 4 patients with benign serous cystadenomas were used for biomarker discovery. Ovarian and patient matched peripheral blood was collected into clotting tubes (Greiner Bio-one, Austria), serum EDTA prepared by centrifugation at 3,000 rpm for 15 min at room temperature and supernatant stored at −80°C.

For the confirmation and validation phases, peripheral plasma samples of patients with high grade serous ovarian carcinomas and normal controls were sourced from the Robinson Research Institute, The University of Adelaide, Adelaide, South Australia; Hudson Institute of Medical Research, Clayton, Victoria, Australia; Department of Obstetrics and Gynecology, Sahlgrenska Cancer Center, University of Gothenburg, Gothenburg, Sweden; Ontario Tumor Bank, Toronto, Ontario, Canada, and Precision Med Inc., Solana Beach, California, USA. Samples for the ovarian cancer subtypes cohort (endometrioid, mucinous and clear cell carcinomas) were obtained from the Robinson Research Institute, The University of Adelaide, Adelaide, South Australia (Table 1).

**Table 1:** Descriptive statistics of patient samples used for the JUP ELISAs.

**Group**	**FIGO stage**	**No**.	**Age**	**CA125 [kU/L]**
**A) MULTICENTRE DATASET - PLASMA EDTA (****FIGURE 3****)**				
High grade serous	IA	18	62 (43–84)	54 (6–11,710)
	IB	2	60 (52–68)	1,247 (730–1,763)
	IC	20	60 (45–86)	232 (17–2,323)
	II	23	60 (41–78)	285 (10–2,446)
	III	34	66 (39–84)	816 (47–11,200)
Normal control	–	109	47 (28–86)	9 (2–54)
**B) OVARIAN CANCER SUBTYPES - SERUM (****FIGURE 4****)**				
Endometrioid	IA	10	58 (41–68)	50 (12–1,564)
	IC	5	55 (33–90)	141 (113–1,856)
Mucinous	IA	9	61 (37–72)	25 (12–1,570)
	IC	3	45 (29–62)	98 (35–126)
Clear cell	IA	9	61 (43–82)	33 (8–593)
	IB	1	52	20
	IC	1	63	20
Normal control	–	39	46 (26–79)	10 (3–63)
**C) BREAST CANCER DATASET - PLASMA EDTA**				
Breast cancer	I	6	52 (41–66)	–
	II	6	61 (46–68)	–
High grade serous	IC	3	50 (45–86)	292 (289–390)
	II	2	58 (53–63)	210 (121–298)
Normal control	–	7	60 (37–72)	13 (5–24)

*Age and CA125 concentrations are given as median with range in brackets. High grade serous stage IC contains one patient without a known CA125 plasma concentration*.

Plasma EDTA samples of early stage breast cancer patients were acquired from Conversant Biosciences Inc., Huntsville, Alabama, USA (Table 1).

### Depletion of Top 14 Abundant Serum Proteins

The 14 most abundant serum proteins were depleted using the multiple affinity removal system (MARS) liquid chromatography (LC) column (Cat. No. 5188-6558, Agilent, Santa Clara, California, USA) according to the manufacturer's recommendations. In brief, 100 μl serum were mixed with 297 μl buffer A (Cat. No. 5185-5987, Agilent) and 3 μl protease inhibitor cocktail (Cat. No. P9599, Sigma-Aldrich, St. Louis, Missouri, USA), then filtered through a 0.22 μm spin filter (Cat. No. CLS8160, Corning, New York, USA) using a centrifuge at 16,000 x g at room temperature for 1 min. Depletion of 160 μl of the filtrate was carried out on an Agilent 1,100 high performance LC (HPLC) using a 4.6 × 100 mm MARS human 14 depletion column. Low abundant protein fraction was collected and mixed 1:5 with 100% (v/v) acetone (ice-cold) and stored at −20°C until further use. Proteins were pelleted by centrifugation for 45 min at 12,000 × g and −9°C. The protein pellet was washed in 3 mL ice-cold 100% (v/v) acetone, stored at −20°C for 1 h then centrifuged for 45 min at 12,000 × g and −9°C. Supernatant was discarded and the protein pellet was air-dried for 10 min to remove residual acetone. Proteins were suspended in 400 μl TUC4% [7 M urea (Cat. No. 1.08487.0500, Merck, Darmstadt, Germany), 2 M thiourea (Cat. No. RPN6301, GE Healthcare, Little Chalfont, UK), 4% (w/v) CHAPS (Cat. No. 10810118001, Roche, Basel, Switzerland), 30 mM Tris (Cat. No. BIO3094T, Astral Scientific, Taren Point, Australia), 1% (v/v) protease inhibitor cocktail (Sigma), 1.1% (v/v) Pefabloc® SC protector reagent (Cat. No. 11873628001, Roche), pH 7.5]. The suspended protein samples were desalted via a 10 kDa cut-off spin filter (Cat. No. VN01H02, Vivacon 500, Sartorius, Göttingen, Germany) by centrifugation for 30 min at 14,000 × g, 15°C. The filter was washed five times by adding 400 μl of TUC4% and centrifugation for 30 min at 14,000 × g, 15°C. The protein concentration of resulting protein sample was measured using an EZQ™ protein quantification kit (Cat. No. R33200, Life Technologies, Carlsbad, California, USA) according to the manufacturer's manual.

### Saturation Labeling Two-Dimensional Difference Gel Electrophoresis (2D DIGE)

Amount of protein labels S-200 and S-300 [Cat. No. PR33, NH DyeAgnostics GmbH, Halle (Saale), Germany] and reduction agent Tris(2-carboxyethyl)phosphine hydrochloride (TCEP) (NH DyeAgnostics) was optimized according to the manufacturer's recommendation and protocols (11) with 1.5 μl of the TCEP (resuspended in 400 μl of H_2_O) and 3 μl of the respective label (S-200 and S-300) chosen for the subsequent saturation DIGE experiment. Six serum samples from the venous flow of malignant stage I epithelial ovarian tumors, the 6 corresponding peripheral serum samples, 6 serum samples from the venous flow of benign ovarian tumors and one internal pooled standard (IPS) were labeled. For this, 5 μg of total proteins from the depleted serum collected from the ovarian venous flow and matched peripheral serum were labeled with S-200. The IPS, consisting of an equal mixture of 5.9 μg of total proteins from each sample was labeled with S-300. Volume of each protein sample was equalized to 9 μl using TUC4%, then proteins were reduced for 1 h at 35°C in the dark using 1.5 μl TCEP for the samples. IPS was diluted with TUC4% to a concentration of 0.56 μg/μl, subsequently 31.9 μl TCEP was added and incubated at 35°C for 1 h in the dark. After reduction, the proteins were labeled using 3 μl of S-200, the IPS was labeled using 63.7 μl of S-300 and incubated for 1 h at 35°C in the dark. For each sample, the reaction was quenched by adding 13.5 μl (IPS: 286.7 μl) of TUC4% supplemented with 2% (w/v) Dithiothreitol (Cat. No. 11583786001, Roche) and 4% (v/v) Pharmalyte 3-10 (Cat. No. 17-0456-01, GE Healthcare).

### 2D PAGE

Eighteen 24 cm immobilized pH gradient (IPG) strips with a pH range of 3-10NL (Cat. No. 1632043, Bio-rad, Hercules, California, USA) were rehydrated in 500 μl of TUC1% (6 M urea, 2 M thiourea, 1.2% (v/v) 2,2 Dithiodiethanol (Cat. No. 380474, Sigma-Aldrich), 0.5% (v/v) Pharmalyte 3-10 (GE Healthcare) overnight at room temperature, then stored at −80°C until further use. 5μg of protein sample mixed with 5 μg IPS was loaded via anodal cup-loading. Isoelectric focusing (IEF) was carried out using an IPGPhor II (GE Healthcare) with following settings: 150 V for 1 h, 300 V for 1 h, 600 V for 1.5 h, increase to 8,000 V by gradient over 2 h, 24.000 Vh at 8,000 V, under exclusion of light. Current was limited to 50 μA per IPG strip. After IEF, IPG strips were stored at −80°C until further use. SDS-PAGE was carried out as described earlier (12).

### Image Acquisition

After SDS-PAGE, gels were scanned using a Typhoon Trio Imager (GE Healthcare). S-200 channel was scanned with a photomultiplier tension (PMT) of 600 V, an emission window of 580 nm BP30, excitation using a green laser (532 nm), S-300 was scanned with a PMT of 600 V, an emission window 670 nm BP30 and excitation with a red laser (633 nm).

### DIGE Data Analysis

Before spot detection, acquired images were warped using Robust Automated Image Normalization (13, 14) applying the recommended standard settings. All gels were aligned to the IPS (S-300 channel) of Gel01 (Figure S1). Protein spot detection was carried out using DeCyder 7.0 (GE Healthcare, RRID: SCR_014592). On average 2,359 (range: 2,174–2,715) spots per gel were detected. The normalized spot volumes were exported using the DeCyder XML toolbox (GE Healthcare), standardized against the spot volume of the corresponding spot in the IPS (S-300) channel and log10 transformed. Data was analyzed using R (version 3.6.2, The R Foundation for Statistical Computing, RRID: SCR_001905) (15) using the additional libraries plyr (16) and reshape (17).

### Liquid Chromatography Tandem Mass Spectrometry (LC-MS/MS)

Primary candidate spots (736, 1,252, 1,295 and 1,507) were excised from eight gels using an Ettan™ SpotPicker (GE Healthcare) and the respective spots were combined. Proteins were digested using Trypsin (Cat. No. V5111, Promega, Madison, Wisconsin, USA). Tryptic peptides were dried using a SpeedVac (Thermo-Fisher, Waltham, Massachusetts, USA) and suspended in 12 μl of FA2 (2% (v/v) acetonitrile (Cat. No. 1.00029.1000, Merck), 0.1% (v/v) formic acid (Cat. No. 5438040100, Sigma-Aldrich)). LC-MS/MS was carried out using an Impact HD mass spectrometer (Bruker Daltonics, Bremen, Germany) as described earlier (18), deviating in applying a flow-rate of 0.3 μl/min and a 70 min gradient for peptide separation. Ten μl of peptide mixture was injected.

### Protein Identification

Acquired mass spectrometry data was converted to Mascot generic format using ProteoWizard version 3.0.20093 (19) and searched against the SwissProt human database (downloaded 03/12/2019; 42,386 entries) using Comet release 2019.01 rev. 4 (20). Precursor mass tolerance was set to 20 ppm, fragment bin tolerance to 0.02. Variable modifications of oxidation of methionine and fixed modification of Cy3 saturation DIGE label on cysteine were specified, with the digestion enzyme specified as trypsin omitting the proline rule with 2 allowed missed cleavages. Peptides with an e-value below 0.05 were regarded as significant, for protein identification only proteins with at least two significant unique peptide identifications were considered. The mass spectrometry proteomics data have been deposited to the ProteomeXchange Consortium via the PRIDE (21) partner repository with the dataset identifier PXD018417. Mass spectrometry data analysis scripts can be downloaded from https://github.com/medardus333/OvCa_Markers.

### Enzyme-Linked Immunosorbent Assay (ELISA)

JUP ELISA kits were purchased from MyBiosource, San Diego, California, USA (Cat. No. MBS2018947). ELISAs were performed according to the manufacturer's protocol with the modification of using 300 μl wash solution (instead of 350 μl). Plate design was randomized using R. Serum or plasma EDTA samples for JUP ELISA were diluted 1:10 in 1 x PBS pH 7.4. ELISA plates were developed for 15 min, afterwards absorbance at 450 nm was measured using a Biotrak II Reader (GE Healthcare). A 4-parameter logistic regression curve was fitted to the absorbance values of the standards for each ELISA plate and used to calculate the JUP concentration of the samples. For the ELISA measuring the JUP concentration in early stage breast cancer, the calibration point with the highest JUP concentration (10 ng/mL) had to be removed from this standard curve as a too low absorbance reading caused the curve fitting to fail. Values between 50 and 100 ng/mL JUP in this ELISA are therefore extrapolated. However, the calibration curves from the earlier conducted JUP ELISA clearly exhibit linear behavior in this range and give precedent to assume a reasonable accuracy of the extrapolated part of the calibration curve used in the early stage breast cancer ELISA (Figure S2). In the multicenter ELISAs, inter-assay coefficient of variance (CV) after calibration using their respective normal control median JUP concentrations was calculated as 6.16% and the median intra-assay CV as 3.96%. For all ELISAs, difference in mean between groups was tested using a two-sided *t*-test, correlations were calculated using a Spearman rank correlation. All analysis steps were performed using R with additional usage of the following packages: drc (22) for calibration curve fitting; ggplot2 (23), ggbeeswarm (24), extrafont (25) and wesanderson (26) for data visualization, dplyr (27), matrixStats (28), and pROC (29) for data analysis and Receiver Operator Characteristics (ROC). The sensitivity at 99.6% specificity level is calculated from a linear regression between the two nearest datapoints spanning 99.6% specificity, the same holds true for specificities at 75% sensitivity in case there was no exact 75% sensitivity datapoint. Positive predictive values (PPV) and negative predictive values (NPV) were calculated using a prevalence of 1:2,500 at a fixed sensitivity level of 75% (4).

## Results

### Saturation 2D DIGE

**Figure 2:**
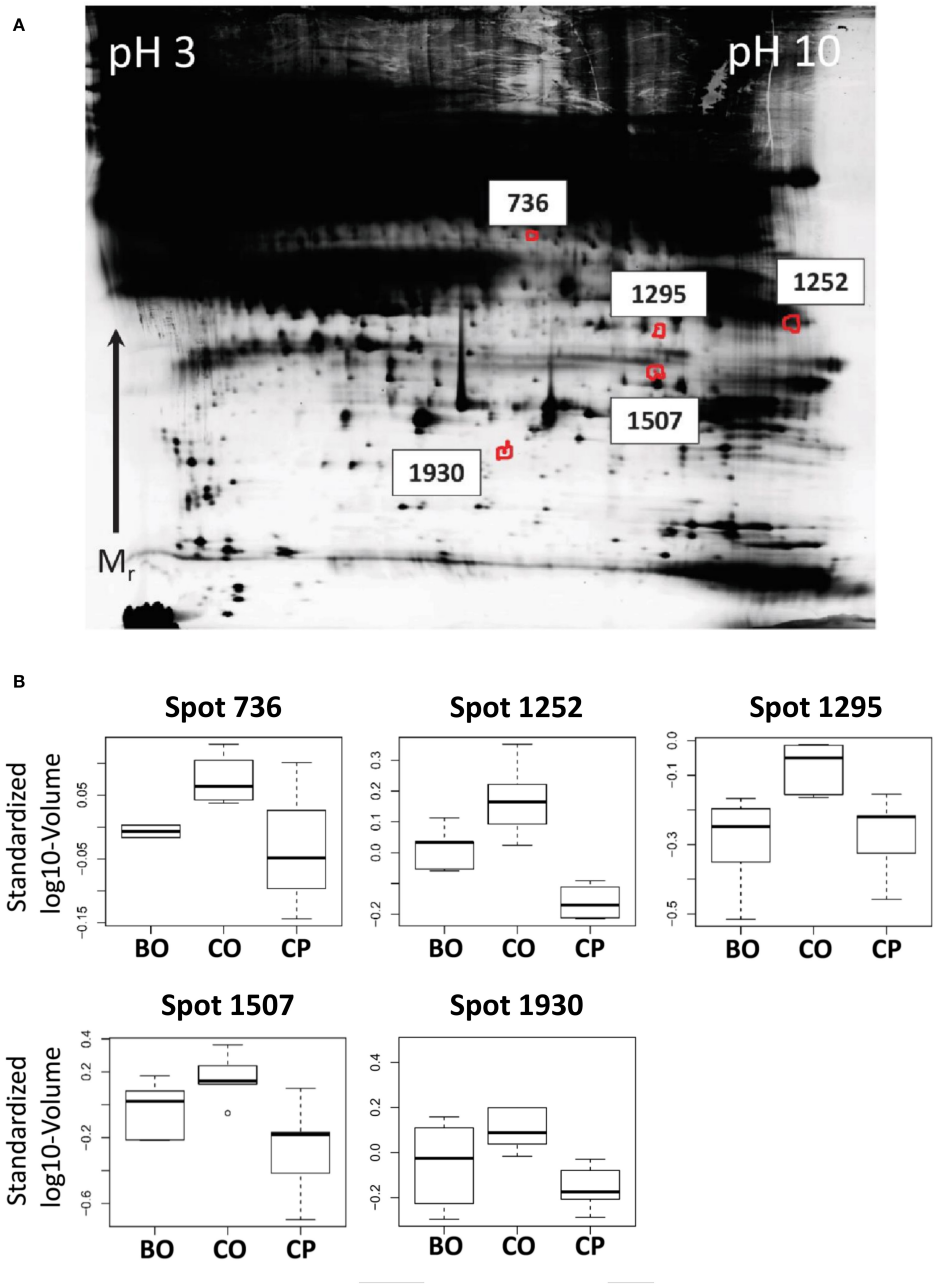
**(A)** Representative, contrast-adjusted DIGE image of depleted serum. Numbers indicate spots higher abundant in ovarian vs. peripheral blood from early stage ovarian cancer patients and ovarian blood from patients with benign ovarian lesions. **(B)** Boxplots of spot abundance across the analyzed DIGE gels. Samples: CO, Cancer ovarian serum; CP, Cancer peripheral serum; and BO, Benign ovarian serum. All *p*-values between CO vs. CP and CO vs. BO < 0.05. JUP was identified in all spots by mass spectrometry (see Table S3).

Our first hypothesis was that proteins which are higher abundant in the venous backflow from early ovarian cancers than in peripheral blood of the same patient are potential markers for early detection. For statistical analysis a paired, one-sided *t*-test was applied. Protein spot volumes exhibiting a *p* < 0.05 and positive fold-change were regarded as significantly higher abundant in ovarian venous backflow. Our second aim was to control for potential increase of protein spot volumes in the ovarian venous backflow due to the surgical procedure. To address this issue, we hypothesized that potential markers for early stage ovarian cancer are higher abundant in venous backflow from malignant tumors than in the venous backflow from benign tumors. This second hypothesis was tested using a one-sided *t*-test. In summary, proteins higher abundant in ovarian serum than in peripheral serum and additionally higher abundant in ovarian serum from malignant tumors than in ovarian serum from benign tumors were regarded as primary candidates for markers of early stage ovarian cancer. Out of a total of 2,692 spots tested, four spots satisfied the outlined criteria (spots 736, 1,252, 1,295, and 1,507, see Figures 2A,B) and were subjected to identification by LC-MS/MS. JUP was identified in all analyzed spots with at least 7 unique peptides (see Table S3) and chosen for further analysis by ELISA.

### JUP Plasma Concentrations Are Increased in Early Stage Serous Ovarian Cancer

The performance of JUP to distinguish patients with early and advanced stage ovarian cancer from normal controls was assessed using an international multicenter patient cohort.

**Figure 3:**
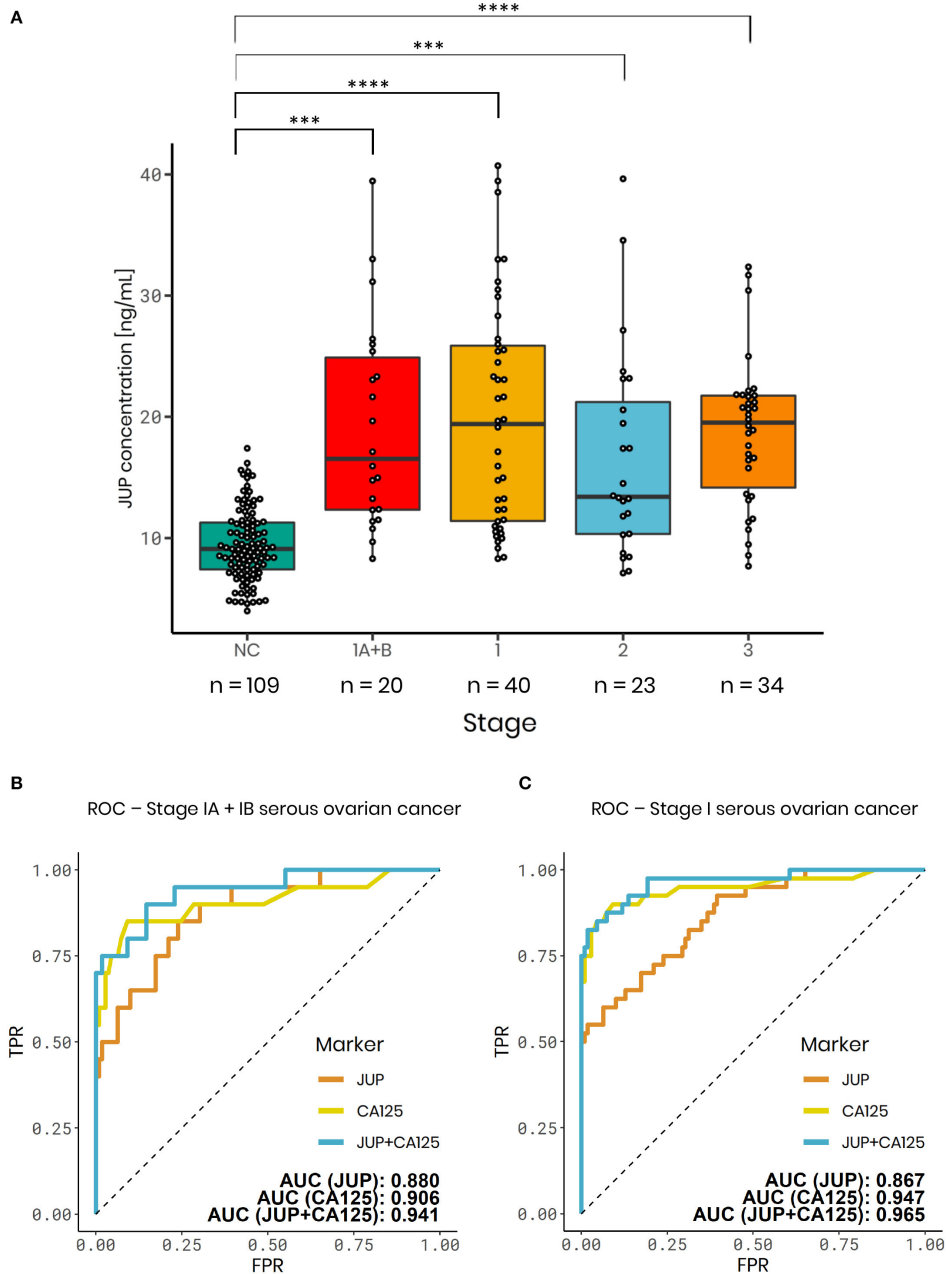
JUP plasma ELISA results for the international, multicenter serous ovarian cancer validation cohort. **(A)** Boxplots of normal controls (NC) (median = 9.09 ng/mL), serous ovarian carcinomas - Stage IA+B (median = 15.45 ng/mL), stage I (median = 18.13 ng/mL), stage II (median = 13.49 ng/mL), and stage III (median = 19.52 ng/mL). Serous carcinomas vs. NC (*n* = 109) - Stage IA+B (1.97-fold change; *p* < 0.001; *n* = 20); stage I (2.09-fold change; *p* < 0.0001; *n* = 40); stage II (1.81-fold change; *p* < 0.001; *n* = 23) and stage III (1.98-fold change; *p* < 0.0001; *n* = 34). **(B,C)** ROC analysis of JUP (orange), CA125 (yellow) and a logistic regression model combining JUP + CA125 (blue). AUC values: **(B)** Serous carcinomas - Stage IA+B: JUP: 0.880; CA125: 0.906; JUP + CA125: 0.941. **(C)** Serous carcinomas - Stage I: JUP: 0.867; CA125: 0.947; JUP + CA125: 0.965. ***p-value < 0.001, ****p-value < 0.0001.

Plasma JUP levels were significantly elevated in serous stage IA+B (1.97-fold increase; *p* < 0.001; *n* = 20), serous stage I (2.09-fold increase; *p* < 0.0001; *n* = 40) and advanced serous stage II (1.81-fold increase, *p* < 0.001, *n* = 23), serous stage III ovarian carcinomas (1.98-fold increase; *p* < 0.0001; *n* = 34) and all stages (1.99-fold-change; *p* < 0.0001, *n* = 97) vs. normal controls (*n* = 109, Figure 3A). To exclude that observed JUP differences between ovarian cancer patients and controls were caused by an origin from different centers, single center subsets of the data were analyzed. JUP plasma concentrations in samples from the Robinson Research Institute were found to be significantly elevated in stage I ovarian cancer (2.45-fold increase; *p* = 0.011; *n* = 8) vs. normal controls (*n* = 82, Figure S4). This was also found in samples from the Sahlgrenska Cancer Center, JUP plasma concentration was elevated in stage IA+B (2.03-fold increase; *p* = 0.016; *n* = 9) and stage I ovarian cancer (1.75-fold increase; *p* = 0.008; *n* = 15) vs. normal controls (*n* = 19; Figure S4). These finding make it very unlikely that our results were confounded by center or selection bias. As next step, the performance of JUP was contrasted against CA125 using ROC. JUP exhibited a similar area under the curve (AUC) as CA125 in stage I (JUP: 0.867; CA125: 0.947) and stage IA+B samples (JUP: 0.880; CA125: 0.906) (Figures 3B,C). ROC for stage II, III, and combined stage I-III is shown in Figure S5. JUP and CA125 were only weakly correlated in ovarian cancer stage I-III (ρ = 0.327) as well as in stage IA+B (ρ = 0.392) and moderately correlated in stage I (ρ = 0.410, data not shown). We therefore established a combined model of JUP and CA125 using logistic regression which resulted in a greater AUC for stage I (0.965) and stage IA+B ovarian cancer (0.941) than for CA125 alone (Figures 3B,C). Sensitivity and specificity levels for all markers and models are displayed in Table 2.

**Table 2:** Sensitivities at a fixed specificity of 100 and 99.6%, specificities at fixed sensitivity of 75% and area under the curve (AUC) of JUP, CA125 and the combination of both markers for stage I–III serous ovarian cancer.

**Model**	**Stage**	**Sensitivity** **(100% specificity)**	**Sensitivity** **(99.6% specificity)**	**Specificity** **(75% sensitivity)**	**AUC**	**PPV**
JUP	IA	0.444	0.444	0.807	0.868	0.002
	IA + IB	0.400	0.422	0.826	0.880	0.002
	IC	0.600	0.600	0.688	0.855	0.001
	I	0.500	0.511	0.761	0.867	0.001
	II	0.391	0.410	0.755	0.818	0.001
	III	0.618	0.656	0.945	0.916	0.005
	I–III	0.515	0.538	0.791	0.873	0.001
CA125	IA	0.500	0.524	0.940	0.896	0.005
	IA + IB	0.550	0.572	0.954	0.906	0.007
	IC	0.800	0.844	1.000	0.989	1.000
	I	0.675	0.708	0.991	0.947	0.032
	II	0.783	0.802	1.000	0.971	1.000
	III	0.971	0.983	1.000	1.000	1.000
	I–III	0.804	0.827	1.000	0.971	1.000
JUP+CA125	IA	0.667	0.667	0.945	0.934	0.005
	IA + IB	0.700	0.700	0.982	0.941	0.016
	IC	0.800	0.844	1.000	0.989	1.000
	I	0.750	0.761	1.000	0.965	1.000
	II	0.783	0.802	1.000	0.970	1.000
	III	1.000	1.000	1.000	1.000	1.000
	I–III	0.804	0.822	1.000	0.976	1.000

*Positive predictive values (PPV) at fixed sensitivity of 75%, based on an ovarian cancer prevalence of 1:2,500 in the population*.

We assessed if JUP plasma concentrations might be age-dependent, as the average age in the control group was slightly lower. However, the correlation analysis of JUP plasma concentrations and age revealed only a weak correlation in normal controls (ρ = 0.188), no correlation in stage I patients (ρ = −0.014) and a weak negative correlation in stage IA+B cases (ρ = −0.382) (Spearman's rank test) (Figure S6).

### JUP Serum Concentration Are Increased in Non-serous Epithelial Ovarian Cancer Subtypes

**Figure 4:**
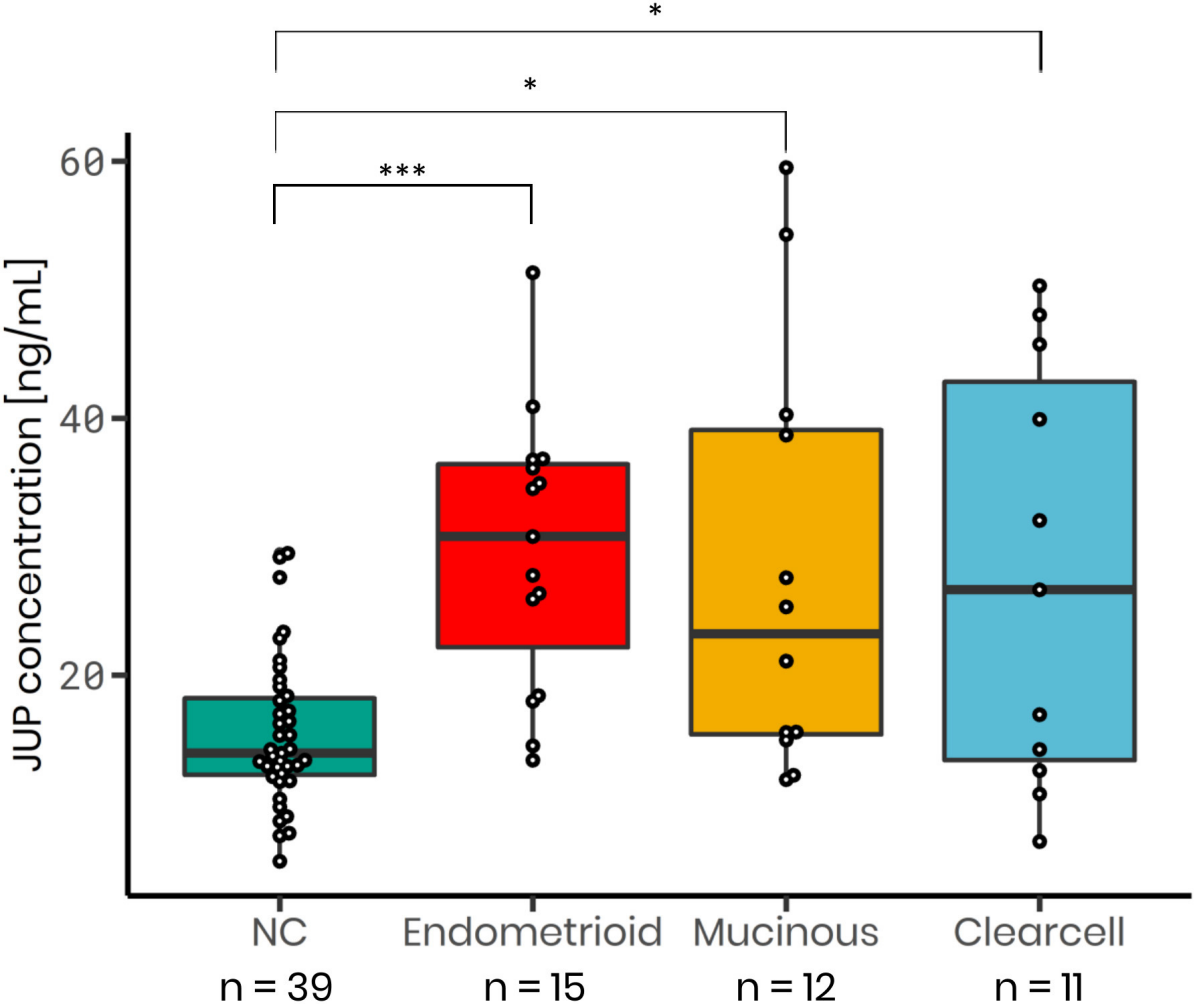
JUP serum concentration is increased in non-serous stage I ovarian cancer subtypes: Endometrioid (median = 30.80 ng/mL; 1.93-fold increase; *p* < 0.001; *n* = 15), mucinous (median = 23.21 ng/mL; 1.82-fold increase; *p* = 0.0229; *n* = 12) and clear cell carcinoma (median = 26.65 ng/mL; 1.79-fold increase; *p* = 0.0329; *n* = 11) as in comparison to normal control samples (NC; median = 13.94 ng/mL; *n* = 39). *P*-values were calculated using a *t*-test. *p-value < 0.05, ***p-value < 0.001.

JUP serum concentration were significantly elevated in stage I non-serous ovarian cancer subtypes such as endometrioid (1.93-fold change; *p* < 0.001; *n* = 15), mucinous (1.82-fold change; *p* = 0.023; *n* = 12) and clear cell carcinomas (1.79-fold change; *p* = 0.033; *n* = 11) versus normal controls (*n* = 39, Figure 4). ROC analysis was conducted to assess the ability of JUP to distinguish between these early stage cancer subtypes and normal controls. In endometrioid carcinomas JUP exhibited an AUC of 0.886, in mucinous carcinomas 0.748 and in clear cell carcinomas 0.690, which is lower than the AUC values of CA125 (endometrioid: 0.925; mucinous: 0.922; clear cell carcinomas: 0.864). As with the serous ovarian cancer samples, we also conducted a logistic regression analysis to determine the effect of the combining JUP and CA125. This resulted in AUC values similar to those with single markers for all tested non-serous subtypes (endometrioid: 0.905; mucinous: 0.923 and clear cell: 0.834) (Figure S7), although there was an increase in AUC for stage IA clear cell carcinoma: JUP: 0.783, CA125: 0.865, JUP+CA125: 0.895 (Figure S7). Sensitivity and Specificity levels for all markers, models and subtypes are shown in Table S8.

### JUP Plasma Concentrations Are Not Increased in Early Stage Breast Cancer

JUP plasma concentrations were not significantly elevated in stage I and II breast cancer (median = 50.4 ng/mL; *p* = 0.122; *n* = 12) when compared to normal controls (median = 36.5 ng/mL; *n* = 7), while JUP plasma levels were significantly increased in stage I and II serous ovarian cancer (median = 68.8 ng/mL; 1.76-fold change; *p* = 0.043; *n* = 5, Figure S9). Further analysis of the breast cancer data was conducted nonetheless as an isolated *p*-value is not a reasonable benchmark for a potential biomarker. ROC analysis was performed and JUP exhibited an AUC of 0.679 in early stage breast cancer (Figure S9).

## Discussion

Ovarian cancer accounts for about 6% of cancer deaths in women and has the highest mortality of all gynecologic malignancies. The best strategy to reduce mortality would be early detection through screening. However, a reliable early detection test which is able to identify the disease at an early, curable state does not exist.

The to-date largest screening trial, the UK Collaborative Trial of Ovarian Cancer Screening (UKCTOCS), employing an algorithm incorporating the CA125 profile over time, failed to reduce ovarian cancer mortality (30). The US Preventative Services Task Force therefore determined that harms of ovarian cancer screening currently outweigh the benefits and advised against ovarian cancer screening in the general population (31).

Numerous efforts have been undertaken to develop a biomarker-based early detection test [reviewed in (32)]. Proteins that are over-expressed by cancer cells and then released into the bloodstream remain the ideal markers for early detection. However, a major “quantitative” challenge of ovarian cancer biomarker research is to identify the cancer at an early stage when it is small, and the amount of cancer-specific proteins secreted into the blood stream is extremely low (5). To overcome this difficulty, we hypothesized that protein markers are higher concentrated in the ovarian venous blood directly downstream of an ovarian cancer than in peripheral blood taken of the cubital vein where the marker is diluted in the total blood volume. The technique of blood sampling from ovarian veins has been reported for endocrinology studies in the past (33, 34) (Figure 1) but to the best of our knowledge, it has never been applied for biomarker discovery in ovarian cancer.

Our strategy resulted in the identification of a promising new marker for epithelial ovarian cancer, JUP. It is primarily considered a protein whose function is to maintain appropriate cell-cell adhesion, but recent studies have also demonstrated that it is involved in signaling and regulation of tumorigenesis as well as cancer progression (35). In ovarian cancer JUP has mainly been described as tumor suppressor inhibiting *in vitro* growth, migration and invasion (36). It has been shown that JUP interacts with both wild type and mutant p53 and its tumor/metastasis suppressor function in some ovarian cancers may, at least partially, be mediated by this interaction (37). However, JUP's role and regulation on the cellular level remains complex, and both positive and negative roles have been found for various malignancies (38).

JUP was proposed as prognostic biomarker in adenocarcinomas of the lung (39) and as tissue biomarker for cervical (neck) lymph node metastasis in oral squamous cell carcinomas (40). Furthermore, JUP was implicated in testicular germ cell tumors (41), and colorectal cancers (42).

Interestingly, a recent study found that high JUP expression enables tumor cells to adhere together and move as clusters into the bloodstream, facilitating metastasis and resulting in worse prognosis in breast cancer patients (43). It is conceivable that a similar process exists for ovarian cancer, where upregulation of JUP contributes to the formation of ovarian cancer cell aggregates known as spheroids, promoting the release of cancer cells into the abdominal cavity.

Due to the low prevalence of ovarian cancer (1 in 2,500 or 40 in 100,000 postmenopausal women) and the strict requirements for a screening strategy, an effective ovarian cancer screening test requires a minimum positive predictive value (PPV) of 10%. To achieve a PPV of 10% with a prevalence of 1 in 2,500, a screening test requires a sensitivity of at least 75% for early stage disease and a specificity of at least 99.6%. As the diagnosis of ovarian cancer generally requires a surgical procedure, a PPV of 10% results in 10 operations for every single case of detected cancer (4). In our study, the combination of JUP and CA125 reached a sensitivity of 75 at 100% specificity for FIGO stage I disease and therefore would fulfill the requirements for an early diagnostic test. JUP plasma concentrations were not elevated in early stage breast cancer patients when compared to normal controls, indicating that JUP might be a specific marker for early stage ovarian cancer.

The strength of our research was the inclusion of a large number of stage I serous ovarian cancer cases and the international multicenter validation approach. Limitations are the retrospective nature of the study with analysis of clinical samples in repositories. A larger prospective study is now required to further validate the utility of the biomarker combination for population screening.

In conclusion, our strategy of analyzing ovarian tumor blood for biomarker discovery identified a novel biomarker, JUP, which in combination with CA125 represents a promising novel diagnostic test for early detection of ovarian cancer.

## Data Availability Statement

The datasets presented in this study can be found in online repositories. The names of the repository/repositories and accession number(s) can be found below: PRIDE via ProteomeXchange (http://www.ebi.ac.uk/pride/archive/projects/PXD018417). FTP download: ftp://ftp.pride.ebi.ac.uk/pride/data/archive/2020/08/PXD018417.

## Ethics Statement

The studies involving human participants were reviewed and approved by Research Ethics Committee at the Royal Adelaide Hospital, Adelaide, South Australia (Protocol #080304). The patients/participants provided their written informed consent to participate in this study.

## Author Contributions

FW contributed to the experimental design, performed the proteomics analysis, ELISAs as well as the data analysis, and wrote the manuscript. NL contributed to the experimental design, performed some of the ELISAs, analyzed data, and revised the manuscript. MK-H performed data analysis, helped writing, and revising the manuscript. TJ, AS, and KS acquired and provided ELISA samples and revised the manuscript. PH and MO conceived the project and wrote the manuscript. MO performed the surgery for ovarian blood sampling. All authors contributed to the article and approved the submitted version.

## Conflict of Interest

The authors declare that the research was conducted in the absence of any commercial or financial relationships that could be construed as a potential conflict of interest.
