# Supplementary material for: Ovarian Blood Sampling Identifies Junction Plakoglobin as a Novel Biomarker of Early Ovarian Cancer
Source: Front Oncol. 2020 Sep 25;10:1767. doi: 10.3389/fonc.2020.01767 (PMC7545354; doi:10.3389/fonc.2020.01767)

Supplementary Figure S1: Saturation labelling DIGE of serum samples – IPG 3-10NL, 24 cm, 5µg protein load, SDS-PAGE: T=12.5%, S-200 channel. -O: Ovarian serum; -P: Peripheral serum. OvCa: Ovarian cancer

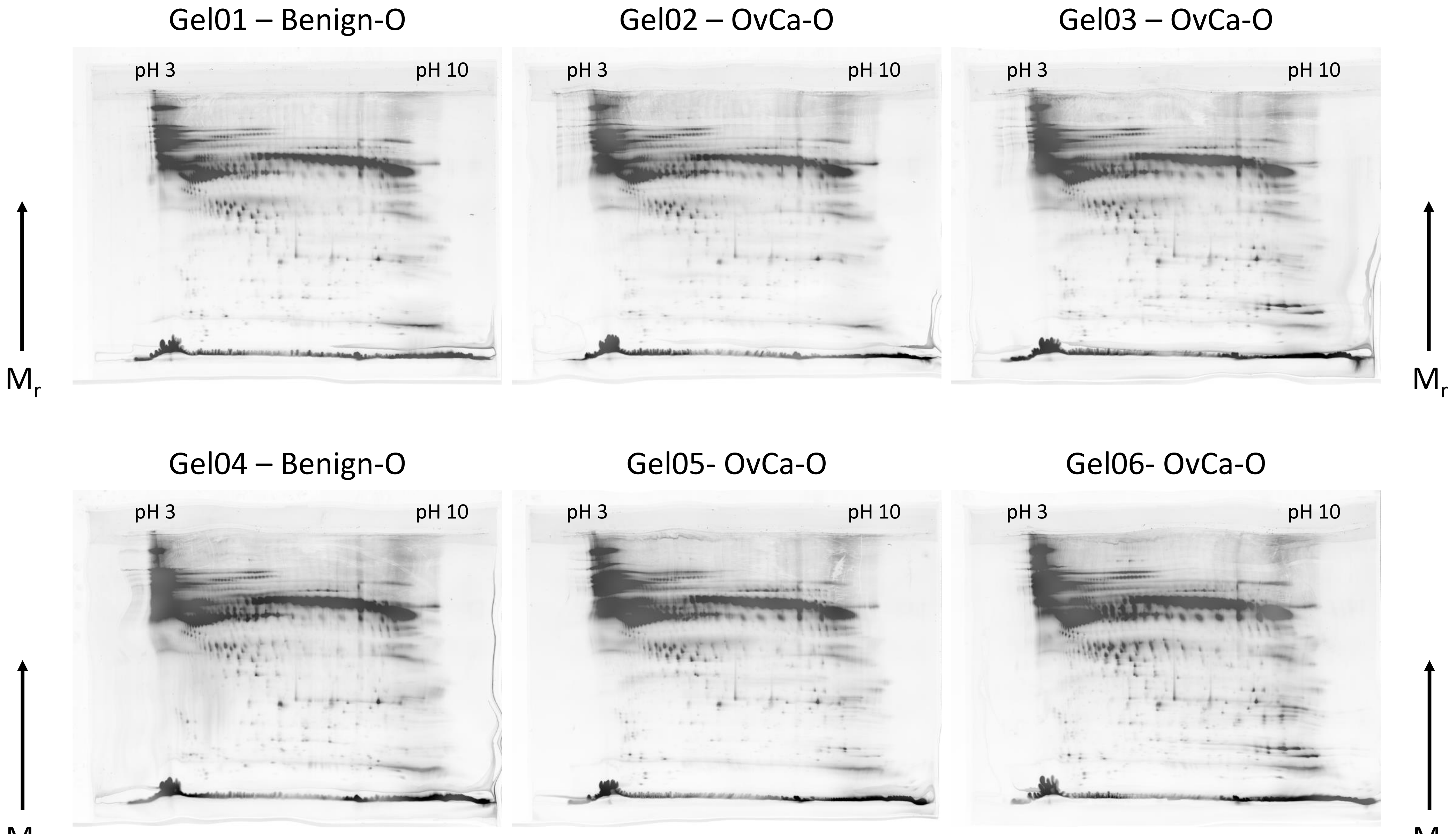

Images after warping with RAIN – not contrast adjusted

Supplementary Figure S1: Saturation labelling DIGE of serum samples – IPG 3-10NL, 24 cm, 5µg protein load, SDS-PAGE: T=12.5%, S-200 channel. -O: Ovarian serum; -P: Peripheral serum. OvCa: Ovarian cancer

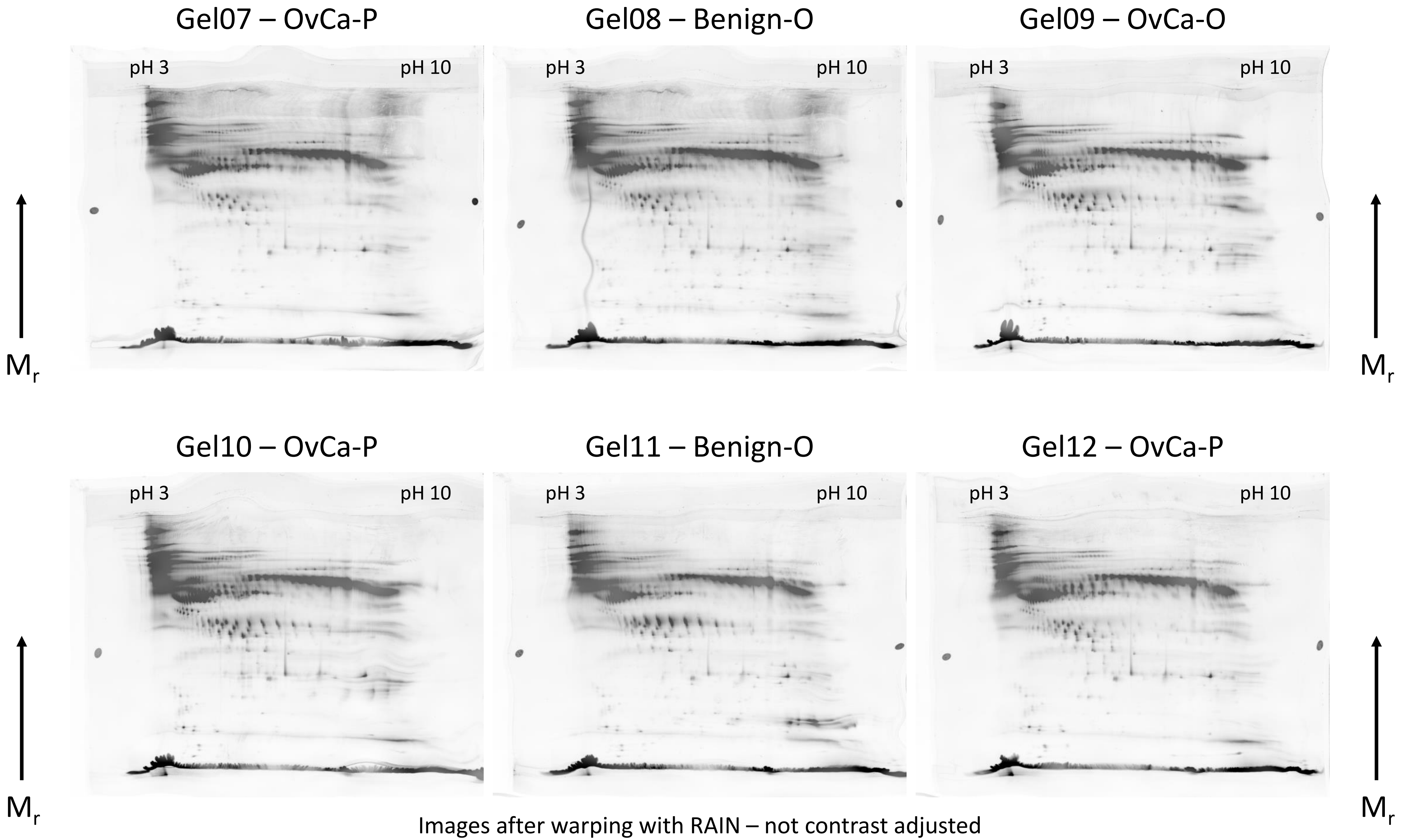

Supplementary Figure S1: Saturation labelling DIGE of serum samples – IPG 3-10NL, 24 cm, 5µg protein load, SDS-PAGE: T=12.5%, S-200 channel. -O: Ovarian serum; -P: Peripheral serum. OvCa: Ovarian cancer

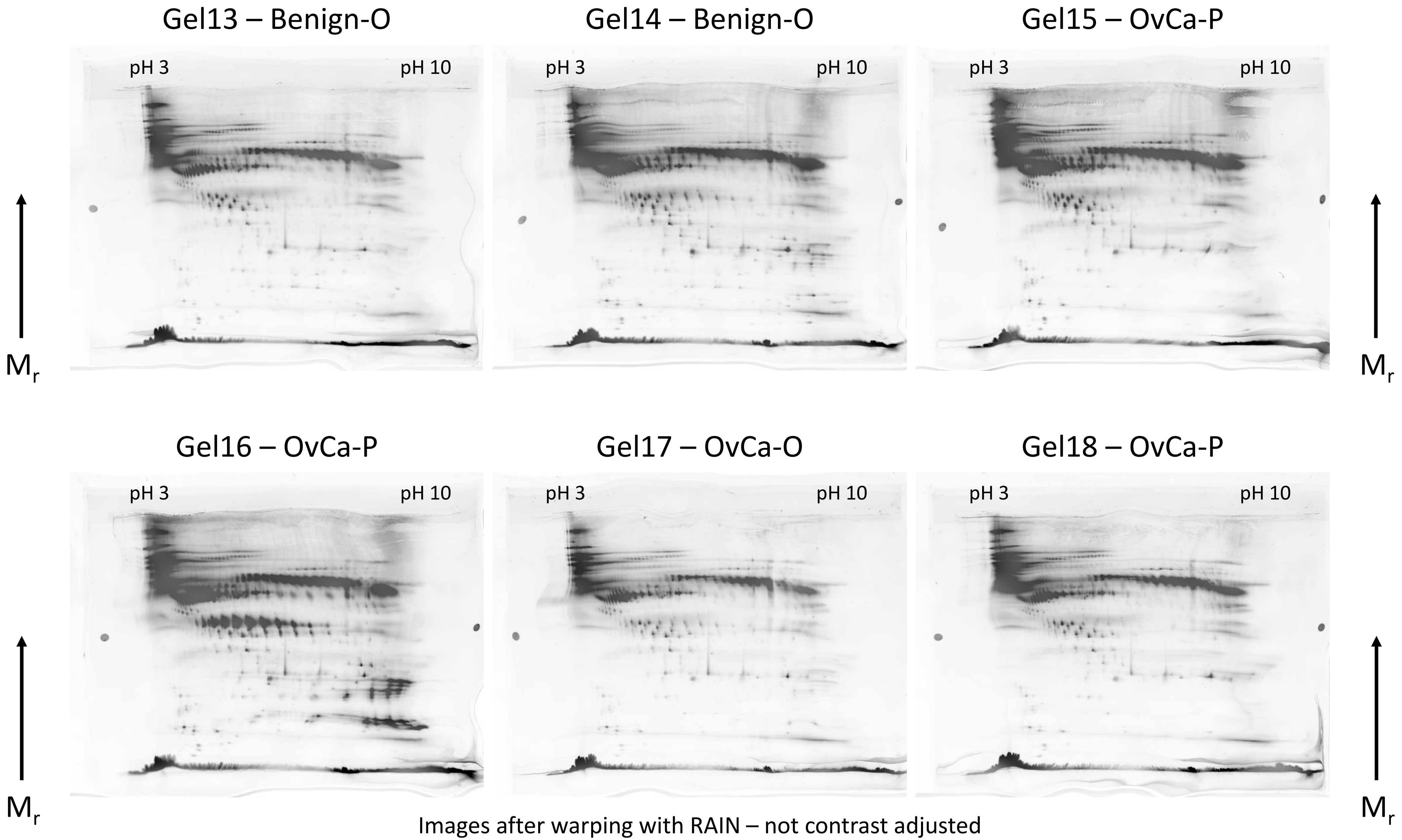

Supplement: Supplementary file 1 [file Image_1.pdf]
