# Supplementary figures and images for: Ovarian Blood Sampling Identifies Junction Plakoglobin as a Novel Biomarker of Early Ovarian Cancer
Source: Front Oncol. 2020 Sep 25;10:1767. doi: 10.3389/fonc.2020.01767 (PMC7545354; doi:10.3389/fonc.2020.01767)

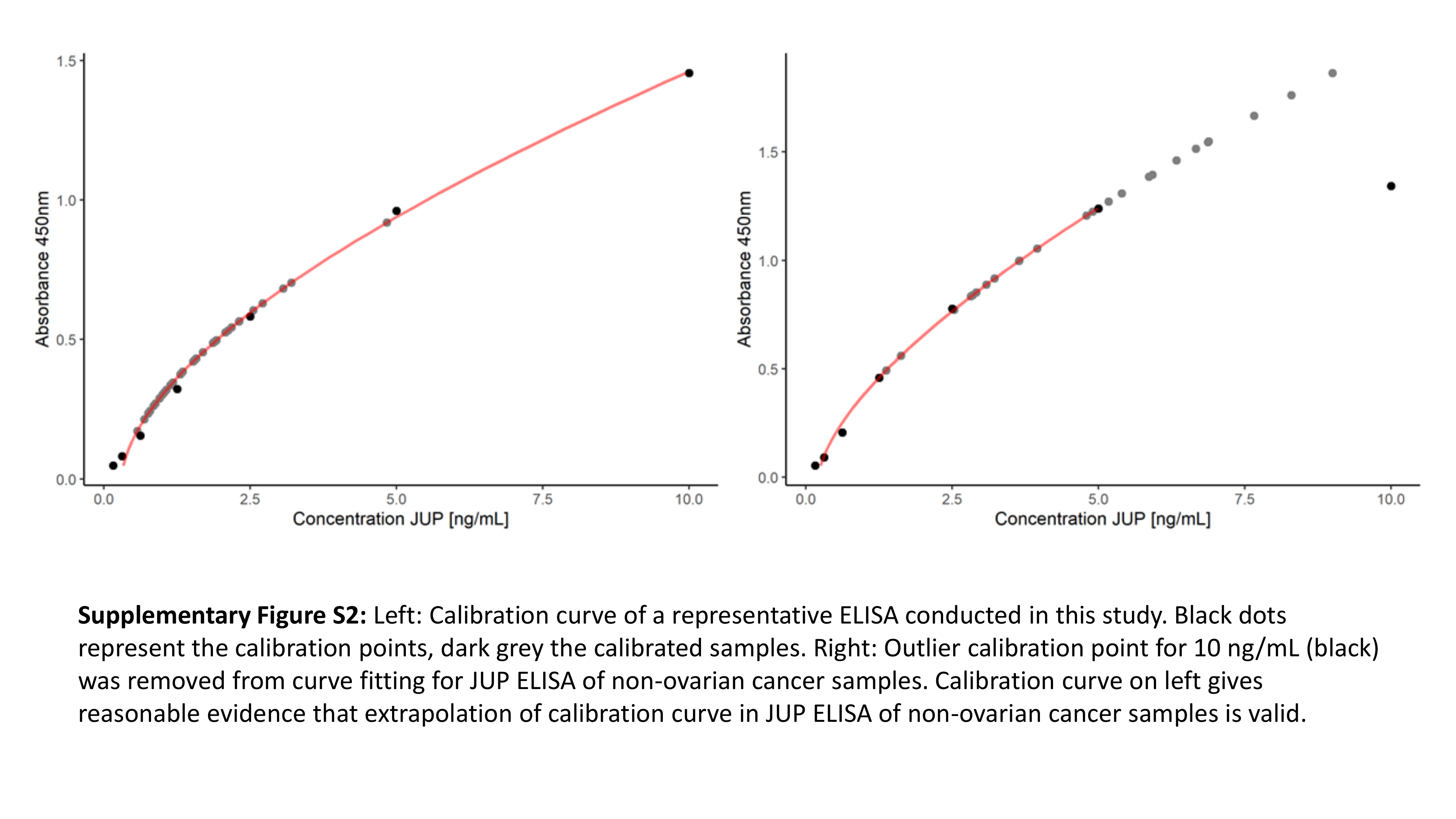

Supplement: Supplementary file 2 [file Image_2.tif]

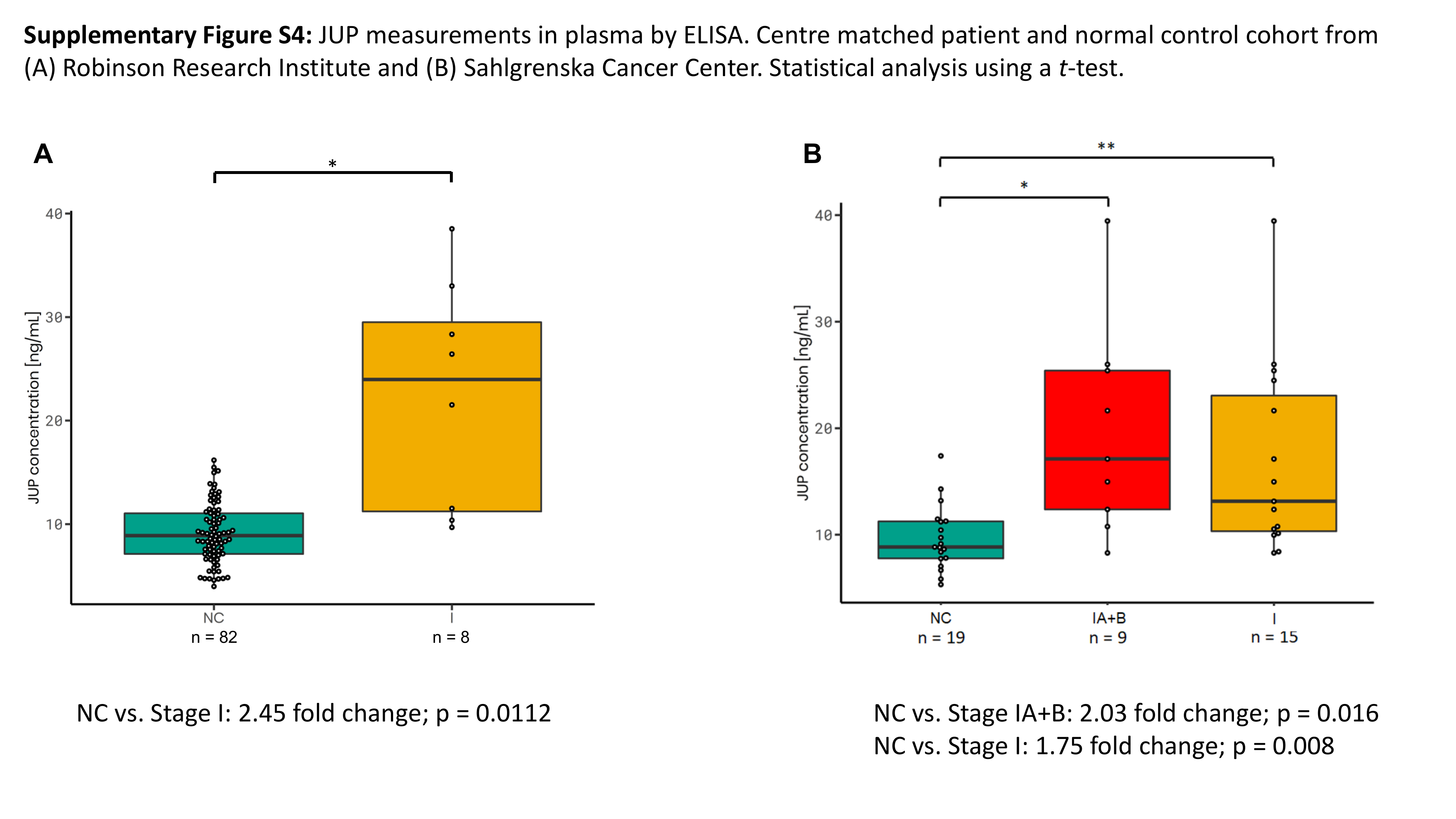

Supplement: Supplementary file 4 [file Image_4.tif]

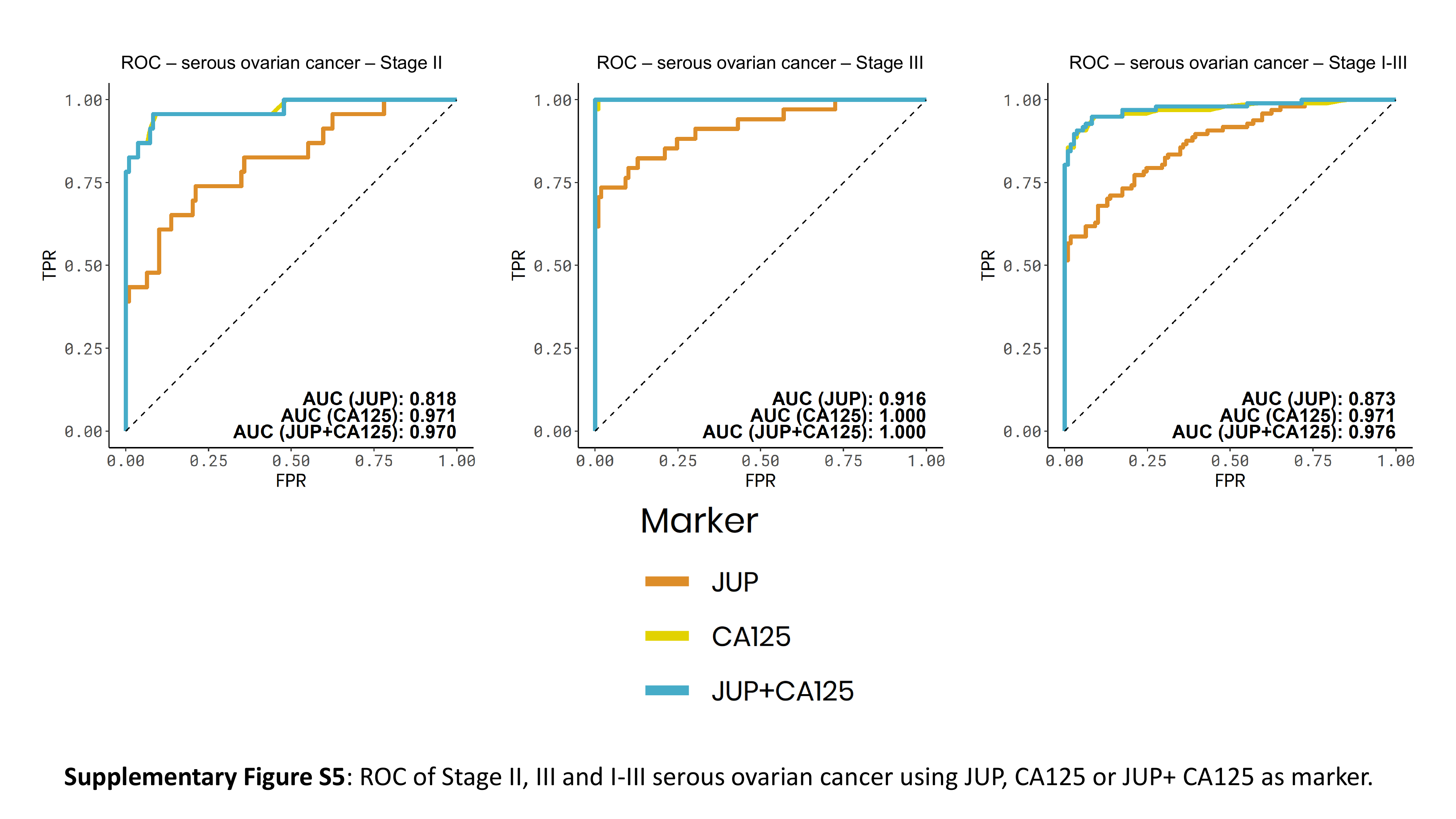

Supplement: Supplementary file 5 [file Image_5.tif]

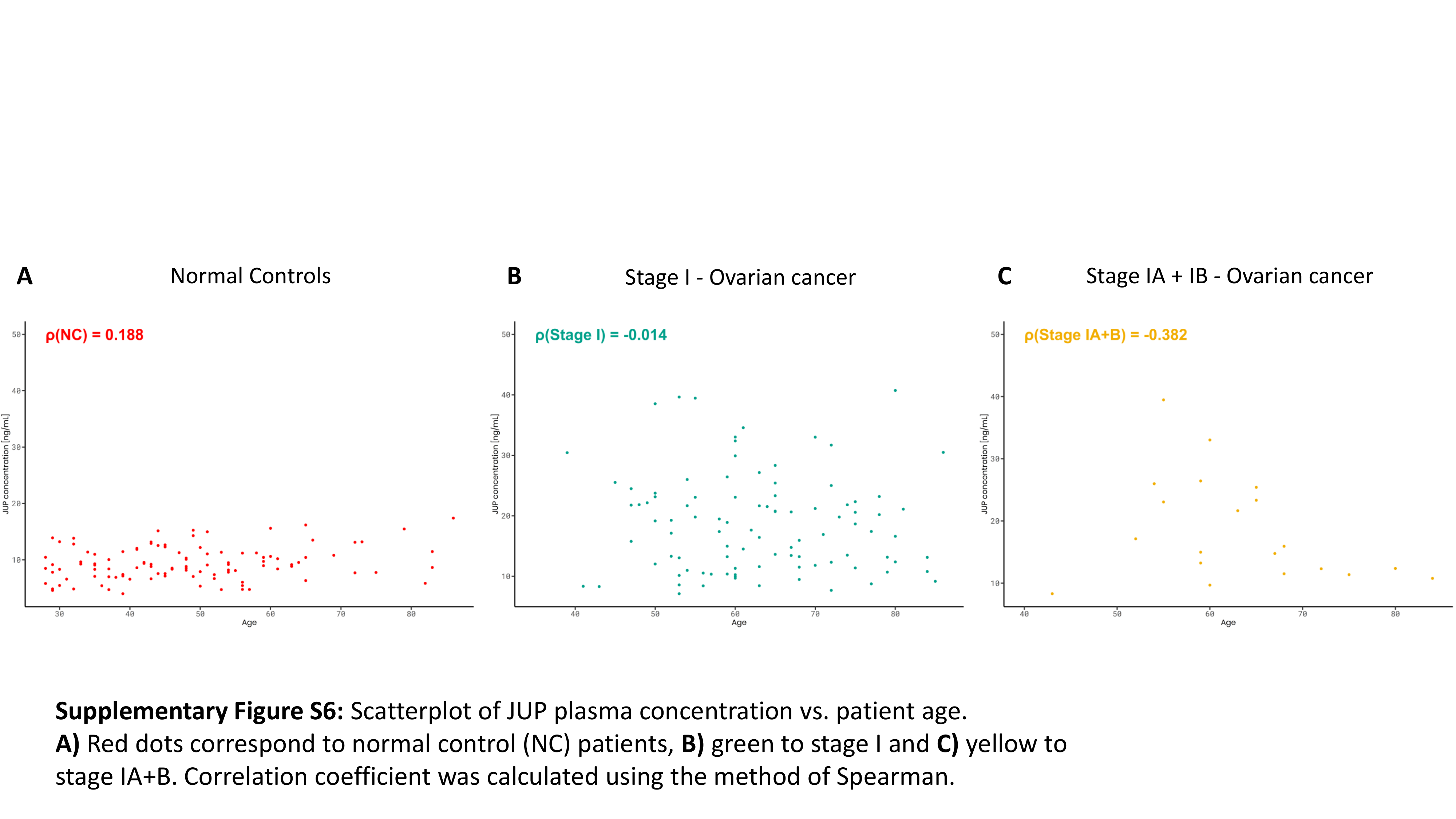

Supplement: Supplementary file 6 [file Image_6.tif]

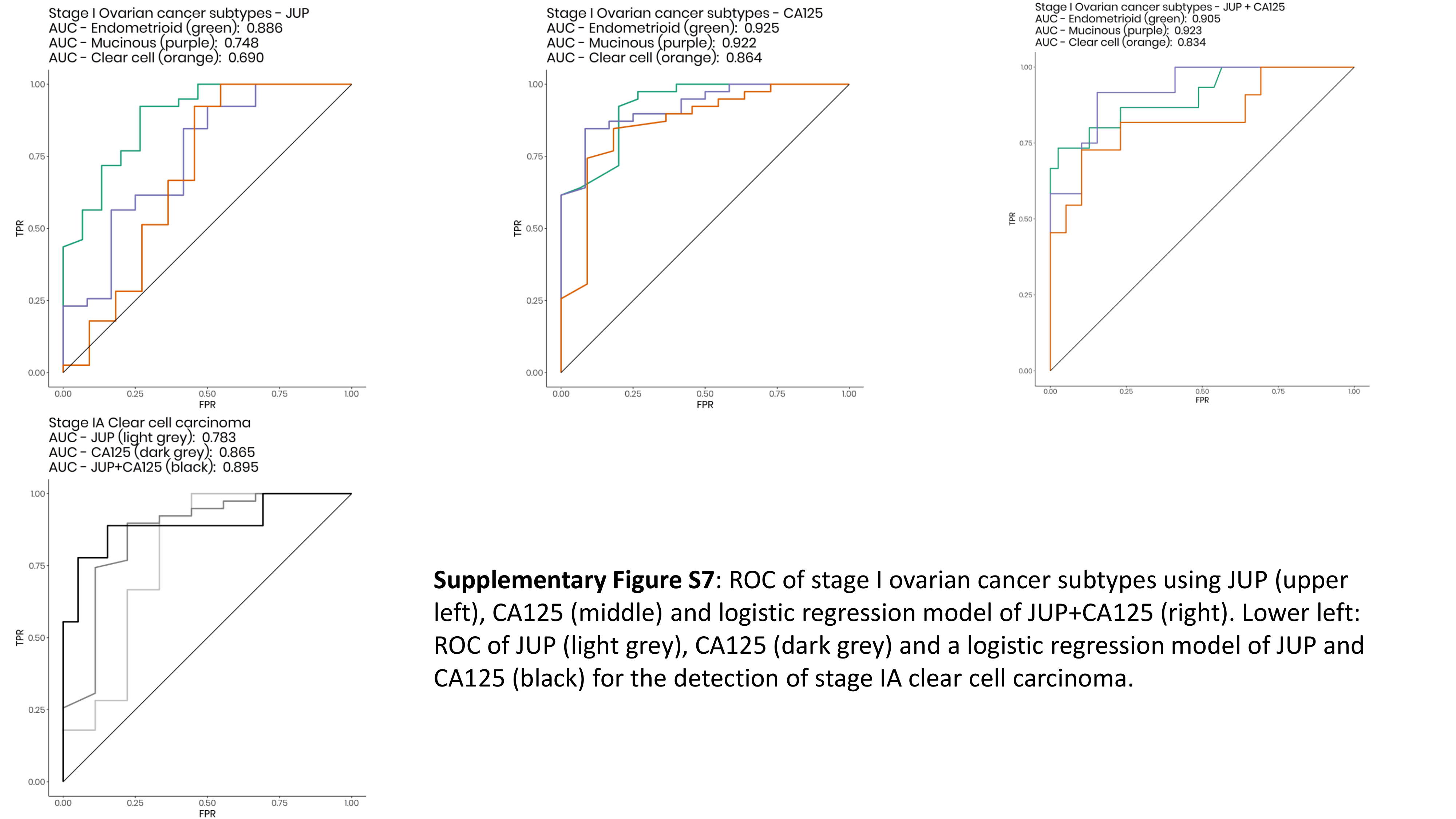

Supplement: Supplementary file 7 [file Image_7.tif]

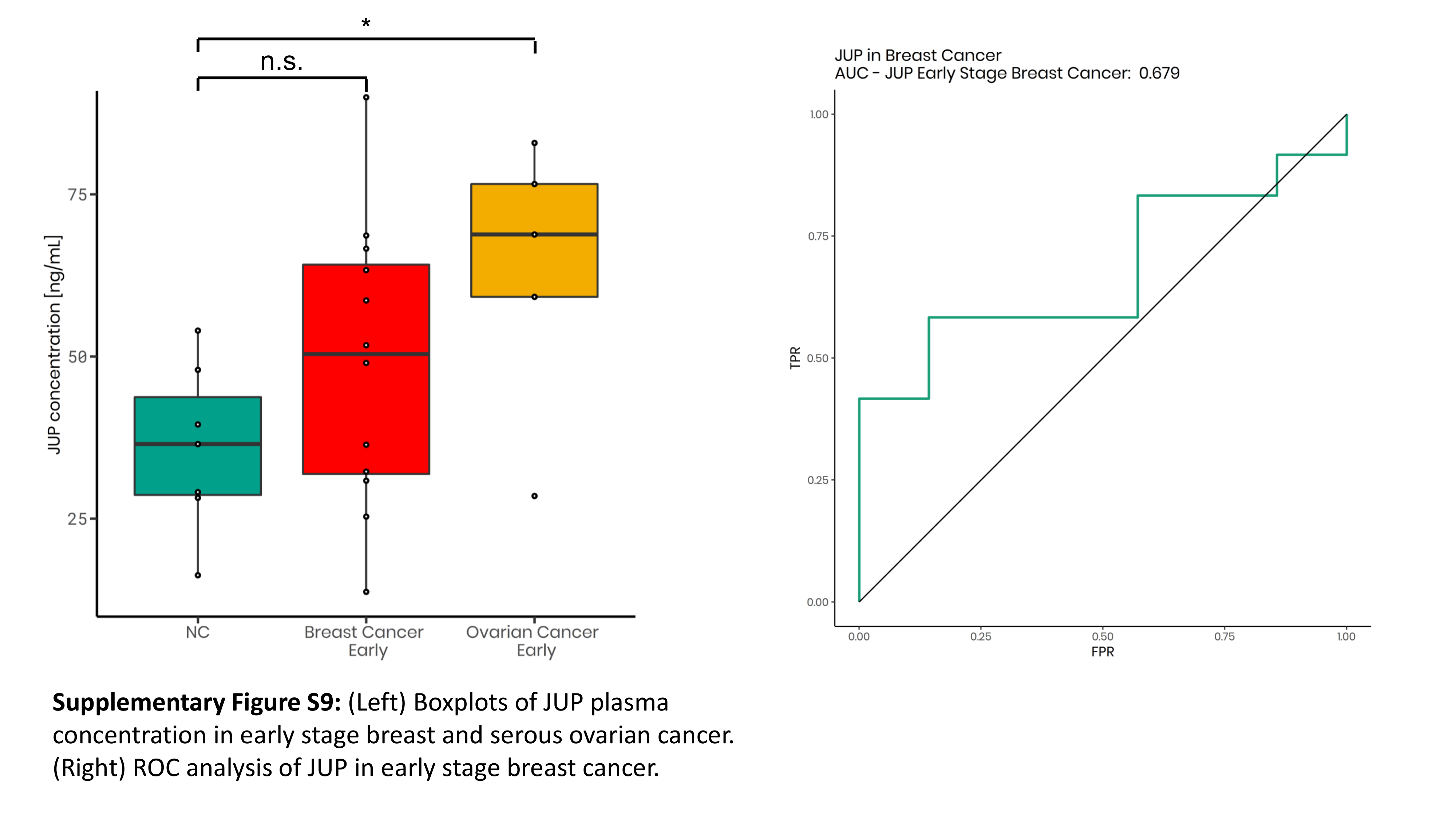

Supplement: Supplementary file 9 [file Image_9.tif]
